# Supplementary material for: KEAP1-Mutant Lung Cancers Weaken Anti-Tumor Immunity and Promote an M2-like Macrophage Phenotype
Source: Int J Mol Sci. 2024 Mar 20;25(6):3510. doi: 10.3390/ijms25063510 (PMC10970780; doi:10.3390/ijms25063510)
Supplement: Supplementary file 1 [file ijms-25-03510-s001.zip › SupplementaryFigures.pptx]

## Slide 1
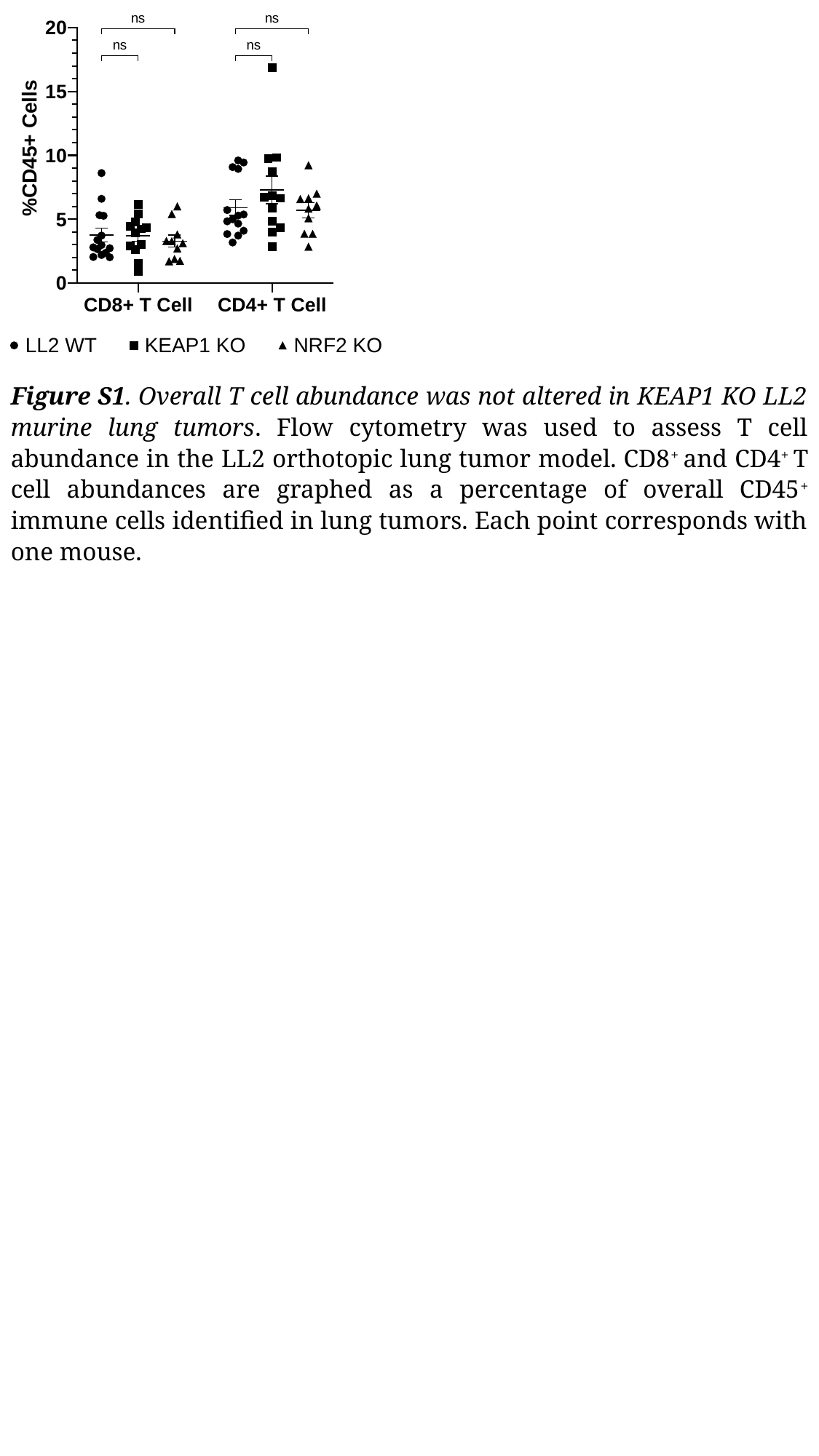

Figure S1. Overall T cell abundance was not altered in KEAP1 KO LL2 murine lung tumors. Flow cytometry was used to assess T cell abundance in the LL2 orthotopic lung tumor model. CD8+ and CD4+ T cell abundances are graphed as a percentage of overall CD45+ immune cells identified in lung tumors. Each point corresponds with one mouse.

## Slide 2
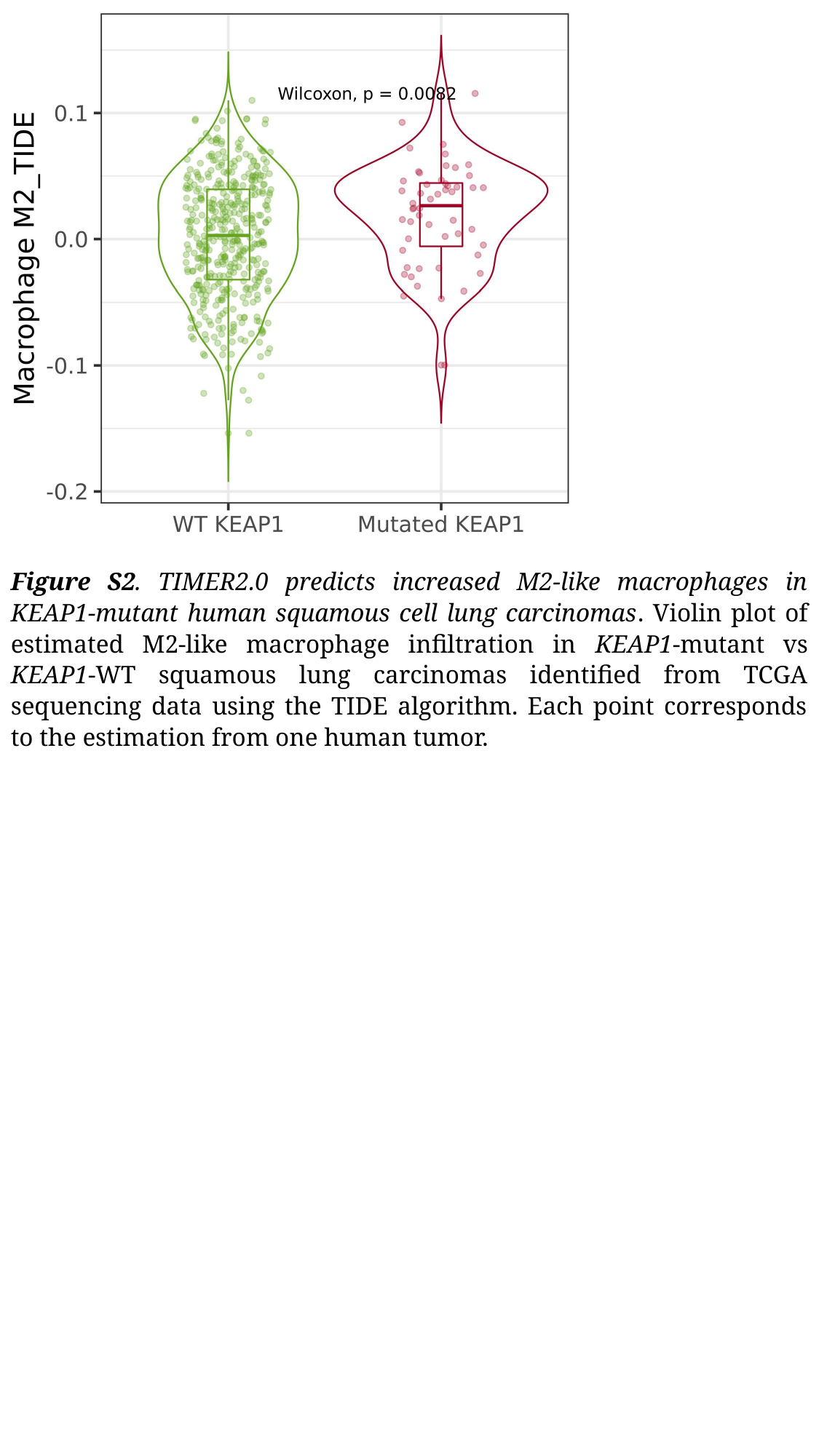

Figure S2. TIMER2.0 predicts increased M2-like macrophages in KEAP1-mutant human squamous cell lung carcinomas. Violin plot of estimated M2-like macrophage infiltration in KEAP1-mutant vs KEAP1-WT squamous lung carcinomas identified from TCGA sequencing data using the TIDE algorithm. Each point corresponds to the estimation from one human tumor.
